# 1064. Treatment Success in Reducing Recurrent *Clostridioides difficile* Infection with Investigational Live Biotherapeutic RBX2660 Is Associated with Microbiota Restoration: Consistent Evidence from a Phase 3 Clinical Trial

**DOI:** 10.1093/ofid/ofab466.1258

**Published:** 2021-12-04

**Authors:** Ken Blount, Dana M Walsh, Carlos Gonzalez, Bill Shannon

**Affiliations:** 1 Rebiotix, Inc., Roseville, Minnesota; 2 BioRankings, LLC, St. Louis, Missouri

## Abstract

**Background:**

Several investigational microbiota-based live biotherapeutics are in clinical development for reducing recurrence of *Clostridioides difficile* infection (rCDI), including RBX2660 a liquid suspension of a broad consortium of microbiota, which includes Bacteridetes and Firmicutes. RBX2660 has been evaluated in >600 participants in 6 clinical trials. Here we report that RBX2660 induced significant shifts to the intestinal microbiota of treatment-responsive participants in PUNCH CD3—a Phase 3 randomized, double-blinded, placebo-controlled trial.

**Methods:**

PUNCH CD3 participants received a single dose of RBX2660 or placebo between 24 to 72 hours after completing rCDI antibiotic treatment. Clinical response was the absence of CDI recurrence at eight weeks after treatment. Participants voluntarily submitted stool samples prior to blinded study treatment (baseline), 1, 4 and 8 weeks, 3 and 6 months after receiving study treatment. Samples were extracted and sequenced using shallow shotgun methods. Operational taxonomic unit (OTU) data were used to calculate relative taxonomic abundance, alpha diversity, and the Microbiome Health Index (MHI)—a biomarker of antibiotic-induced dysbiosis and restoration.

**Results:**

Clinically, RBX2660 demonstrated superior efficacy versus placebo (70.4% versus 58.1%). From before to after treatment, RBX2660-treated clinical responders’ microbiome diversity shifted significantly (Mann-Whitney), and so did microbiome composition (Generalized Wald Test). Post-treatment changes were characterized by increased Bacteroidia and Clostridia and decreased Gammaproteobacteria and Bacilli, changes and were durable to at least 6 months. Repeated measures analysis confirmed that shifts were greater among RBX2660 responders compared to placebo responders (DMRepeat). The majority of responders’ MHI values shifted from a range common to antibiotic dysbiosis to a range common in healthy populations.

Figure 1

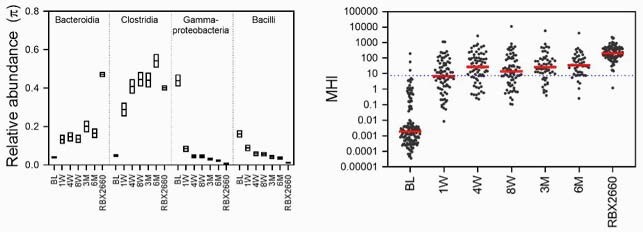

Left panel. Mean relative abundance taxonomic class level at timepoints for participants in PUNCH CD3 before and after RBX2660 treatment, and for doses of RBX2660 administered in PUNCH CD3. The four taxonomic classes that change most from before to after treatment are shown with the mean and confidence intervals based on fitting OTU data to a Dirichlet multinomial distribution. Right panel, MHI biomarker for the same time points and investigational product groups, shown as median (red) and individual samples. A previously calculated threshold of MHI = 7.2 is shown (dotted line), above which MHI values predict healthy, below which MHI values predict antibiotic-induced dysbiosis.

**Conclusion:**

Among PUNCH CD3 clinical responders, RBX2660 significantly restored microbiota from less to more healthy compositions, and this restoration was durable to at least 6 months. These clinically-correlated microbiome shifts are highly consistent with results from multiple prior trials of RBX2660.

**Disclosures:**

**Ken Blount, PhD**, **Rebiotix Inc., a Ferring Company** (Employee) **Dana M. Walsh, PhD**, **Rebiotix** (Employee)

